# The economic burden of overweight and obesity in Saudi Arabia

**DOI:** 10.1371/journal.pone.0264993

**Published:** 2022-03-08

**Authors:** Jesse D. Malkin, Drishti Baid, Reem F. Alsukait, Taghred Alghaith, Mohammed Alluhidan, Hana Alabdulkarim, Abdulaziz Altowaijri, Ziyad S. Almalki, Christopher H. Herbst, Eric Andrew Finkelstein, Sameh El-Saharty, Nahar Alazemi

**Affiliations:** 1 World Bank Group Consultant, Colorado Springs, Colo., United States of America; 2 Sol Price School of Public Policy, University of Southern California, Los Angeles, Calif., United States of America; 3 Community Health Sciences, College of Applied Medical Sciences, King Saud University, Riyadh, Saudi Arabia; 4 Health, Nutrition and Population Global Practice, World Bank, Riyadh, Saudi Arabia; 5 Saudi Health Council, Riyadh, Saudi Arabia; 6 Lancaster University, Lancaster, United Kingdom; 7 Drug Policy and Economic Centre, Ministry of National Guards Health Affairs, Riyadh, Saudi Arabia; 8 Program for Health Assurance and Purchasing, Ministry of Health, Riyadh, Saudi Arabia; 9 Department of Clinical Pharmacy, College of Pharmacy, Prince Sattam Bin Abdulaziz University, Al-Kharj, Saudi Arabia; 10 Duke-NUS Medical School, Singapore, Singapore; 11 Health, Nutrition and Population Global Practice, World Bank, Kuwait City, Kuwait; The Ohio State University College of Medicine, UNITED STATES

## Abstract

**Context:**

The prevalence of overweight and obesity in Saudi Arabia has been rising. Although the health burden of excess weight is well established, little is known about the economic burden.

**Aims:**

To assess the economic burden—both direct medical costs and the value of absenteeism and presenteeism—resulting from overweight and obesity in Saudi Arabia.

**Settings and design:**

The cost of overweight and obesity in Saudi Arabia was estimated from a societal perspective using an epidemiologic approach.

**Methods and materials:**

Data were obtained from previously published studies and secondary databases.

**Statistical analysis used:**

Overweight/obesity-attributable costs were calculated for six major noncommunicable diseases; sensitivity analyses were conducted for key model parameters.

**Results:**

The impact of overweight and obesity for these diseases is found to directly cost a total of $3.8 billion, equal to 4.3 percent of total health expenditures in Saudi Arabia in 2019. Estimated overweight and obesity–attributable absenteeism and presenteeism costs a total of $15.5 billion, equal to 0.9 percent of GDP in 2019.

**Conclusions:**

Even when limited to six diseases and a subset of total indirect costs, results indicate that overweight and obesity are a significant economic burden in Saudi Arabia. Future studies should identify strategies to reduce the health and economic burden resulting from excess weight in Saudi Arabia.

## Introduction

The prevalence of overweight and obesity is high among the population of Saudi Arabia. According to the 2019 Kingdom of Saudi Arabia World Health Survey—the latest nationally representative survey that includes anthropometric measurements of adults—the prevalence of overweight [Body Mass Index (BMI) 25.0 kg/m^2^ to <30 kg/m^2^] is 38 percent and the prevalence of obesity (BMI ≥ 30 kg/m^2^) is 20 percent [1]. The median age of the Saudi population is 31.8 years [1]; obese Saudis who are young are at increased risk for obesity-attributable diseases that are more likely to occur in mid- and late life.

Obesity is a well-established risk factor for all-cause mortality and noncommunicable diseases (NCDs) such as hypertension, dyslipidemia, type 2 diabetes, stroke, cardiovascular diseases, osteoarthritis, several cancers and other conditions. Even people who are overweight but not obese are at increased risk for several of these conditions [2, 3]. This increase in risk translates into substantially higher health care utilization and medical costs among those with excess weight.

Much of the increase in health care utilization and costs results from treating the medical conditions such as type 2 diabetes that are caused by excess weight. However, cost increases also result from direct treatments for obesity, including weight loss surgeries and medications, which are increasingly available in Saudi Arabia. In addition to direct medical costs, excess weight leads to an increase in indirect costs, including increased absenteeism (workdays missed due to illness or injury), presenteeism (reduced productivity while working), reduced labor force participation, and premature mortality. Other, less tangible costs include the monetary value of pain and suffering and opportunity costs resulting from lower economic output.

Much has been written about the economic costs of overweight and obesity in North America [4, 5], Europe [6–11], Brazil [12], Australia [13], and China [14]. These studies used a range of approaches, including regression analyses, epidemiological methods, and simulations. By contrast, we identified just one study that attempted to quantify the economic burden of overweight and obesity in Saudi Arabia [15]. This study included more than 50 countries and contained many assumptions required for comparability across countries.

Given the paucity of research on the costs of overweight and obesity in Saudi Arabia, the objective of this study was to provide estimates of direct and indirect costs using the best available data from within the country. This limitation required us to use an epidemiological approach and to limit the analysis to costs resulting from six major NCDs. Using these diseases we quantified (1) direct medical costs, including the cost of hospitalizations, outpatient visits, emergency department visits, general practitioner visits, and prescription drugs; and (2) indirect costs arising from absenteeism and presenteeism only.

### Subjects and methods

We used a population attributable fraction (PAF) approach—an epidemiologic method widely used to assess the public health impact of exposures in populations—to estimate costs. Direct medical, absenteeism, and presenteeism costs resulting from excess weight in Saudi Arabia in 2019 were quantified for the six NCDs for which data were available: coronary heart disease, stroke, type 2 diabetes, breast cancer in women, colon cancer, and asthma. We excluded NCDs for which data were not available such as major depressive disorder, anxiety disorders, Alzheimer’s, epilepsy, sleep disorders, and several cancers.

We began by obtaining risk ratios—the proportionate increase in risk as a result of overweight or obesity, respectively—for each of the six NCDs. We used sex-specific risk ratio estimates from a previous published study [2], whose results were based upon a meta-analysis of the literature. Our approach assumes that (a) the relative risk from excess weight for these six diseases is the same for Saudi Arabians as it is for those from other countries and (b) the relative risk from excess weight for these diseases has not changed over time.

Next, we calculated the sex-specific PAFs for overweight and obesity, respectively, using the following formulas:

$$PAF_{1}(\%)=\frac{p_{1}(RR_{1}-1)}{1+p_{1}(RR_{1}-1)+p_{2}(RR_{2}-1)}$$

$$PAF_{2}(\%)=\frac{p_{2}(RR_{2}-1)}{1+p_{1}(RR_{1}-1)+p_{2}(RR_{2}-1)}$$

where p_1_ and p_2_ represent the prevalence of overweight and obesity in the population of interest (that is, Saudi Arabia’s population) respectively; RR_1_ represents the unadjusted relative risk of a particular NCD of interest for overweight relative to normal weight (18.5 kg/m^2^ ≤ BMI < 25 kg/m^2^) individuals; and RR_2_ represents the corresponding figure for obesity relative to normal weight individuals. We obtained sex-specific prevalence estimates for overweight and obesity from the 2019 Kingdom of Saudi Arabia World Health Survey [1].

We obtained estimates of the total direct cost, absenteeism cost, and presenteeism cost of the six diseases in Saudi Arabia for the year 2019 from a previous published study [16]. We obtained sex-specific prevalence rates for the year 2019 from the Global Burden of Disease database [17].

To estimate direct medical costs of overweight and obesity, we multiplied the sex-specific prevalence rate by total cost then divided the product by the all-sex prevalence rate to obtain sex-specific total cost estimates. We then multiplied the sex-specific direct medical cost estimates by PAFs to obtain sex-specific estimates of overweight/obesity-attributable direct medical costs. We summed the sex-specific overweight/obesity-attributable direct medical cost estimates for each disease (i.e., direct medical costs for men plus direct medical costs for women) to obtain overweight/obesity-attributable cost estimates for both sexes in the aggregate.

To estimate absenteeism and presenteeism costs of overweight and obesity, we first calculated a weighted average of PAFs for males and females, where the average was weighted by the percentage of the workforce that is male (84.2 percent) or female (15.8 percent), respectively. The breakdown of men and women in Saudi Arabia’s workforce was obtained from The World Bank’s Databank [18]. Next, we multiplied the weighted PAF by total absenteeism or presenteeism costs, respectively, to obtain estimates of overweight- and obesity-attributable costs for both sexes in the aggregate.

All cost estimates are reported in 2019 International dollars.

To test the variation of our results to our assumptions, we conducted several sensitivity analyses. We considered the impact of replacing the base case per unit type 2 diabetes mellitus cost estimates (based on a previous published study [19]) with lower and higher per unit cost estimates from published studies conducted in Saudi Arabia [20, 21]. We replaced base case type 2 diabetes prevalence data from the IHME’s Global Burden of Disease database [17] with higher prevalence estimates from the International Diabetes Foundation [22].

## Results

Estimated costs due to overweight and obesity are presented in Table 1. The estimated annual direct medical cost of excess weight (overweight and obesity combined) is $3.8 billion, equal to 4.3 percent of health expenditures in Saudi Arabia and 0.1 percent of GDP in 2019. Estimated costs of overweight/obesity-attributable absenteeism and presenteeism are $1.6 billion (0.1 percent of GDP in 2019) and $13.8 billion (0.8 percent of GDP in 2019), respectively. Type 2 diabetes is by far the largest cost driver, accounting for 88 percent of overweight/obesity-attributable direct medical costs and 83 percent of overweight/obesity-attributable absenteeism and presenteeism costs.

**Table 1:** Estimated costs attributable to overweight and obesity.

**a. Overweight only**														
	**Relative risk of overweight** ^**a**^		**Population Attributable Fraction** ^**b**^		**Sex-specific prevalence** ^**c**^		**Total direct medical costs** ^**d**^^,^ ^**e**^	**Sex-specific total direct medical costs** ^**d**^		**Total costs attributable to overweight** ^**d**^				
										**Direct medical**			**Absenteeism**	**Presenteeism**
	Males	Females	Males	Females	Males	Females		Males	Females	Male	Female	All	All	All
Coronary heart disease	1.29	1.80	0.10	0.16	2.98%	1.68%	798	511	287	51	46	97	166	404
Stroke	1.23	1.15	0.08	0.04	1.28%	1.56%	1,118	504	614	40	25	65	63	153
Type 2 diabetes	2.40	3.92	0.22	0.23	7.78%	6.46%	4,796	2,620	2,176	576	500	1,077	286	4,074
Breast cancer	n.a.	1.08	n.a.	0.03	0.00%	0.32%	43	0	43	0	1	1	1	1
Colorectal cancer	1.51	1.45	0.16	0.12	0.06%	0.03%	45	29	16	5	2	7	12	15
Asthma	1.20	1.25	0.07	0.07	2.54%	6.46%	352	99	253	7	18	25	130	286
All	n.a.	n.a.	n.a.	n.a.	n.a.	n.a.	7,152	3,763	3,389	679	592	1,271	658	4,932
**b. Obese only**														
	**Relative risk of obesity** ^**a**^		**Population Attributable Fraction** ^**b**^		**Sex-specific prevalence** ^**c**^		**Total direct medical costs** ^**d**^^,^ ^**e**^	**Sex-specific total direct medical costs** ^**d**^		**Total costs attributable to obesity** ^**d**^				
										**Direct medical**			**Absenteeism**	**Presenteeism**
	Males	Females	Males	Females	Males	Females		Males	Females	Male	Female	All	All	All
Coronary heart disease	1.72	3.10	0.11	0.25	2.98%	1.68%	798	511	287	56	72	128	201	487
Stroke	1.51	1.49	0.08	0.09	1.28%	1.56%	1,118	504	614	40	55	96	70	169
Type 2 diabetes	6.74	12.41	0.41	0.54	7.78%	6.46%	4,796	2,620	2,176	1,074	1,175	2,249	555	7,916
Breast cancer	n.a.	1.13	n.a.	0.02	0.00%	0.32%	43	0	43	0	1	1	1	1
Colorectal cancer	1.95	1.66	0.13	0.10	0.06%	0.03%	45	29	16	4	2	5	10	12
Asthma	1.43	1.78	0.07	0.13	2.54%	6.46%	352	99	253	7	33	40	148	325
All	n.a.	n.a.	n.a.	n.a.	n.a.	n.a.	7,152	3,763	3,389	1,182	1,337	2,519	984	8,910
**c. Overweight and obesity**														
	**Total costs attributable to both overweight and obesity** ^**e**^													
	**Direct medical**			**Absenteeism**	**Presenteeism**	**Absenteeism + Presenteeism**								
	Males	Females	All	All	All	All								
Coronary heart disease	107	118	225	367	891	1,257								
Stroke	81	80	160	133	322	455								
Type 2 diabetes	1,651	1,675	3,326	841	11,990	12,831								
Breast cancer	n.a.	2	2	2	2	4								
Colorectal cancer	8	4	12	22	26	48								
Asthma	14	51	64	278	611	888								
All	1,861	1,929	3,790	1,642	13,842	15,484								
% of health expenditures in 2019	2.1	2.2	4.3	n.a.	n.a.	n.a.								
% of GDP in 2019	0.1	0.1	0.2	0.1%	0.8%	0.9%								

n.a. = not applicable.

^a^ Relative risks of comorbidities—the proportionate increase in risk as a result of overweight or obesity—were obtained from Guh, Zhang, Bansback et al. 2009 [2]. We attributed all overweight/obesity-attributable breast cancer costs to women.

^b^ Population attributable fractions (PAFs) for overweight and obesity are calculated using the formula: *PAF*_*1*_ (%) = [*p*_*1*_(*RR*_*1*_ − 1)]/ [1 + *p*_*1*_(*RR*_*1*_ − 1) + *p*_*2*_(*RR*_*2*_ − 1)] and *PAF*_*2*_ (%) = [*p*_*2*_(*RR*_*2*_ − 1)]/ [1 + *p*_*1*_(*RR*_*1*_ − 1) + *p*_*2*_(*RR*_*2*_ − 1)], respectively, where 1 and 2 represent the overweight and obesity groups, respectively; *p* = prevalence rate; *RR* = relative risk. Prevalence estimates for overweight (Male: 43%, Female: 33%) and obesity (Male: 19%, Female: 20%) were obtained from preliminary findings for the 2019 Kingdom of Saudi Arabia World Health Survey [1].

^c^ Global Burden of Disease database [17].

^d^ Millions of 2019 International dollars.

^e^ Finkelstein, Malkin, Baid et al. [16].

The results of our sensitivity analyses are shown in Table 2. When we replace our base case per-unit cost estimates for type 2 diabetes with lower estimates from the literature [20], estimated medical costs decline to $3.2 billion, which is 3.7 percent of estimated health expenditures in 2019. When we replace our base case per-unit cost estimates for type 2 diabetes with higher estimates from the literature [21], estimated direct medical costs attributable to overweight and obesity balloon to $15.9 billion, equal to 18.3 percent of estimated health expenditures in 2019. When we replace our base case estimate of type 2 diabetes prevalence with upper bound estimates from the literature [22], estimated direct medical costs due to overweight and obesity rise to $6.2 billion (7.1 percent of health expenditures in 2019), estimated overweight/obesity-attributable absenteeism costs rise slightly to $2.3 billion (0.1 percent of GDP in 2019) and estimated overweight/obesity-attributable presenteeism costs rise to $22.5 billion (1.3 percent of GDP in 2019).

**Table 2:** Sensitivity analyses.

	Estimates of costs arising from overweight and obesity ^a^					
	Direct Medical		Absenteeism		Presenteeism	
	$	% of health expenditures in 2019	$	% of GDP in 2019	$	% of GDP in 2019
Base case	3,790	4.3	1,642	0.1	13,842	0.8
Higher per-unit diabetes costs [20]	15,943	18.3	*	*	*	*
Lower per-unit diabetes costs [19]	3,222	3.7	*	*	*	*
Higher diabetes prevalence [21]	6,203	7.1	2,252	0.1	22,541	1.3

An asterisk (*) indicates no change from the base case.

^a^ All cost estimates are presented in millions of 2019 International dollars.

## Discussion

Our estimates, albeit limited to six diseases and estimated with great uncertainty, reveal the substantial economic toll of overweight and obesity in Saudi Arabia. Despite limiting our analysis to a small number of NCDs, our base case estimate of annual direct medical costs due to overweight and obesity is $3.8 billion annually, which represents 4.3 percent of total health spending in 2019. The estimated impact of overweight and obesity on absenteeism and presenteeism is even higher: We estimate that overweight and obesity raise absenteeism and presenteeism costs in the aggregate by $15.5 billion annually—equal to 0.9 percent of GDP in 2019.

Our analysis, however, is subject to several important limitations:

- Due to data constraints, we excluded some NCDs for which overweight and obesity-attributable costs may be significant.
- The literature provides a wide range of estimates of the per unit cost and prevalence of type 2 diabetes, which is by far the largest driver of overweight/obesity-attributable costs among the diseases we considered.
- As noted in the introduction, given the paucity of available information, the objective of this study was to provide estimates of direct and indirect costs of overweight and obesity using the best available data. This limitation required us to use an epidemiological approach and stratifications by gender only. We could not, for example, quantify the extent to which costs differ for additional population subsets, such as stratifications by employment characteristics, as this level of detail was not available in the existing data. If such data become available more detailed stratifications are recommended.

- We were unable to capture some kinds of indirect costs such as early retirement and care provided by family and friends.
- The PAF approach assumes that, conditional on diagnosis, treatment and costs do not vary by baseline BMI. If for example, individuals with excess weight are more difficult to treat, then our results would be conservative.

We took into account some but not all of these uncertainties in our sensitivity analyses.

As noted above, we identified only one previous study estimating the cost of overweight and obesity in Saudi Arabia. Cecchini and Vuik (2019) [15] estimate that overweight and obesity will increase Saudi Arabia’s annual health expenditures by 12.7 percent on average between the years 2020 and 2050. Compared to studies in other countries, this estimate is an outlier. The rest of the literature spanning Europe, North America, Australia, China, and Brazil indicates that overweight/obesity-attributable direct medical costs are equal to between 2 and 9 percent of total health expenditures [4–14]. Our base case estimate for Saudi Arabia, although limited to 6 diseases, reveals that overweight/obesity-attributable direct medical costs are equal to 4.3 percent of national health expenditures, which is in the range of previous estimates.

Cecchini and Vuik (2019) [15]—taking into account the effect of excess weight on absenteeism, presenteeism, unemployment, and early retirement—estimate that overweight and obesity will reduce annual GDP in Saudi Arabia by 4.4 percent on average between 2020 and 2050. This, too, is an outlier compared to prior studies. Other researchers have estimated indirect costs resulting from overweight/obesity in France, Australia, Germany, and Canada ranging from 0.5 percent to 1.6 percent of GDP [5, 6, 8, 10, 13]. Our base case estimate of absenteeism and presenteeism costs arising from overweight and obesity in Saudi Arabia—0.9 percent of GDP—is in between these prior estimates.

### Conclusion

Despite uncertainties, our estimates are broadly consistent with previous estimates of the costs of overweight and obesity in countries other than Saudi Arabia. Our findings indicate that overweight and obesity are a significant economic burden in Saudi Arabia, resulting in estimated direct medical costs of 4.3 percent of annual health expenditures and select indirect costs accounting for an estimated 0.9 percent of GDP. This raises the possibility that interventions that prevent overweight and obesity, such as Saudi Arabia’s excise tax on sugar-sweetened beverages [23], may be both health improving and cost saving.
